# Effect of the ethinylestradiol/norelgestromin contraceptive patch on body composition. Results of bioelectrical impedance analysis in a population of Italian women

**DOI:** 10.1186/1475-2891-7-21

**Published:** 2008-08-26

**Authors:** Antonio Piccoli, PierGiorgio Crosignani, Carmine Nappi, Salvatore Ronsini, Vincenzina Bruni, Silvia Marelli, the Italian EVRA Contrast Study Group

**Affiliations:** 1 Department of Medical and Surgical Sciences, University of Padova, Italy; 2 II Institute of Obstetrics and Gynaecology, University of Milan, Italy; 3 Department of Obstetric – Gynaecological, Urological Sciences and Reproduction Medicine, University of Naples, Italy; 4 Department of Maternal and Children's Sciences ASL SA 3, San Luca Hospital, Vallo della Lucania (SA), Italy; 5 Department of Gynaecology, Perinatology and Human Reproduction University of Florence, Italy; 6 Medical Affairs Department, Janssen-Cilag SpA, Cologno Monzese (MI), Italy

## Abstract

**Background:**

As weight gain is one of the most frequently cited reasons for not using and for discontinuing hormonal contraceptives, in an open-label, single-arm, multicentre clinical study we evaluated the effect of the ethinylestradiol/norelgestromin contraceptive patch (EVRA, Janssen-Cilag International, Belgium) on body composition using bioelectrical impedance analysis (BIA).

**Methods:**

Body weight and impedance vector components (resistance (R) and reactance (Xc), at 50 kHz frequency, Akern-RJL Systems analyzer) were recorded before entry, after 1, 3 and 6 months in 182 Italian healthy women aged 29 yr (18 to 45), and with BMI 21.8 kg/m^2 ^(16 to 31). Total body water (TBW) was estimated with a BIA regression equation. Vector BIA was performed with the RXc mean graph method and the Hotelling's T^2 ^test for paired and unpaired data.

**Results:**

After 6 months body weight increased by 0.64 kg (1.1%) and TBW increased by 0.51 L (1.7%). The pattern of impedance vector displacement indicated a small increase in soft tissue hydration (interstitial gel fluid). Body composition changes did not significantly differ among groups of previous contraceptive methods. Arterial blood pressure did not significantly change over time.

**Conclusion:**

After 6 months of treatment with the ethinylestradiol/norelgestromin contraceptive patch we found a minimal, clinically not relevant, increase in body weight less than 1 kg that could be attributed to an adaptive interstitial gel hydration. This fluctuation is physiological as confirmed by the lack of any effect on blood pressure. This could be useful in increasing women's choice, acceptability and compliance of the ethinylestradiol/norelgestromin contraceptive patch.

## Background

The efficacy and safety of the ethinylestradiol/norelgestromin contraceptive patch compared to monophasic and triphasic oral contraceptives have been demonstrated in controlled, open-label pivotal studies, assessing 3,319 subjects for a total of 22,160 cycles [1].

A large open-label, single-arm, multicentre clinical study (Phase IIIB/IV, NRGEEP-CON-402 "EVRA Contrast") has been carried out in Europe to evaluate women's experience with the ethinylestradiol/norelgestromin contraceptive patch, specifically, user satisfaction and user preference for the patch compared with their previous method of contraception. The open study design with broad selection criteria enabled the closest possible reflection of a routine clinical setting. Results will be published in a different paper. A group of participant investigators from Italy amended the study protocol including, as secondary objectives, an additional sub-study to assess body composition with bioelectrical impedance analysis (BIA), whose results will be presented in this paper.

BIA is a non invasive method specific for assessment of soft tissue hydration based on the effect of the current flow through intra- and extracellular ionic solutions. The impedance vector Z is a combination of resistance (R) (i.e. the opposition to flow of an alternating current through intra and extracellular ionic solutions) and reactance (Xc) (i.e. the capacitative component of tissue interfaces, and cell membranes and organelles). The arc tangent of Xc/R is called the phase angle. Measurements of Z vector are used for assessment of total body water (TBW) either through BIA regression equations or with Vector BIA [2-5]. Contribution of bone to impedance is negligible, and lean soft tissue contributes more than fat because adipocyte droplets of triacylglycerols are non-conductors. Although body impedance reflects tissue hydration, soft tissue mass (lean and fat) can also be empirically derived by correlation in healthy subjects because the compartments of soft tissue are correlated with each other through physiological constants. Physiological constants become flawed in patients with fluid disorders, which accounts for most conflicting results of literature [5]. An advantage of the vector approach is the lack of reliance on regression models to predict TBW and the inherent error associated with the use of group models to predict individual TBW [4,5].

The effect of hormonal contraceptives on body weight gain is still debated. Weight gain is often considered one of the most frequently cited side effects of using combined contraceptives and many women and clinicians believe that an association exists [6]. In a random survey conducted in the UK, almost 75% of women reported that weight gain was related to oral contraceptive use [7]; moreover, in one national study in the United States, 60% of pill users who returned to their healthcare provider due to side effects were concerned about weight gain [8]. In Europe, about 30% of women surveyed from representative populations in Germany and France claimed to have gained weight on their pills [9,10]. Concern about weight gain limits the use of this highly effective method of contraception, especially in younger women, and can cause early discontinuation or poor compliance among users. Even the mere perception of weight gain, increased body fat or fluid overload can lead to contraceptive discontinuation. A national representative sample of American women indicated that weight gain was the most common single reason for discontinuing oral contraception: at 11% this was a more frequent reason than nausea, headache and menstrual abnormalities [11]. A survey of 6676 women from several European countries similarly found that weight gain was among the most common complaints and was associated with a 40% increased likelihood of discontinuation [12]. However, a recent meta-analysis did not find evidence supporting a causal association between combination oral contraceptives or combination skin patch and weight gain. Authors concluded that available evidence was insufficient to determine the effect of combination contraceptives on weight, but no large effect was evident [13]. Published studies with the contraceptive patch (one pooled analysis of 3 clinical trials, one placebo controlled trial and one comparative trial versus a triphasic contraceptive pill) confirm that users of the patch generally experience minimal changes in body weight. For example, in the comparative trial of the patch versus a pill containing levonorgestrel 50/75/125 μg + ethinylestradiol 30/40/30 μg, the mean increase in body weight was 0.41 kg in both treatment groups and the distribution of users who gained or lost weight or remained within 5% of baseline weight during the trial was comparable in the two groups [14]. However, as in real life weight gain, perception of increased body fat or water retention and fear of putting on weight remain big concerns that can either deter the initiation of hormonal contraception or cause early discontinuation among users, we think that the objective evaluation and monitoring of body composition may contribute to improve the acceptability of estroprogestin methods to women. Therefore, even if some studies did not show a significant increase in body weight with the contraceptive patch, it is a challenge for body composition analysis to determine if changes occur in some compartments.

In this study we used BIA methods to evaluate body composition changes following the use of the ethinylestradiol/norelgestromin contraceptive patch for 6 months.

## Methods

### Study design

This was a multicentre (24 Italian centres), open-label, single-arm clinical study with treatment duration of 6 months, i.e. 6 treatment cycles of 4 weeks. Each site's Independent Ethics Committees (IECs) approved the study protocol and related amendments prior to the start of the study, which was conducted, between June 2004 and November 2005, in accordance with the Declaration of Helsinki. Each ethinylestradiol/norelgestromin contraceptive patch (EVRA: a three layers 20 cm^2 ^contraceptive patch which contains ethinylestradiol 0.6 mg and norelgestromin 6.0 mg and delivers a daily dose of ethinylestradiol 20 μg and norelgestromin 150 μg over the 7 days period – Janssen-Cilag International, Belgium) was worn for one week and was replaced on the same day of the week for three consecutive weeks. The fourth week was "patch-free".

Four clinic visits were scheduled: a screening visit (T0), followed by visits after one month (Cycle 1, T1), after 3 months (Cycle 3, T3) and after 6 months (Cycle 6, T6) with the ethinylestradiol/norelgestromin contraceptive patch.

At T0, the subject was asked about her method of contraception used in the three months before entering the study. Compliance was assessed at all visits by inspection of returned study medication boxes and review of Diary Cards in which the subject recorded the dates and sites of patch applications and details in the case of any patch detachments. A perfect compliance cycle was defined as 21 consecutive days of patch use with no patch worn longer than 7 days and the patch-free interval minimally 1 and maximally 7 days. A subject compliant score (%) was calculated as number perfect compliant cycles/number ITT cycles per each woman.

Height was recorded at T0. Subjects were weighed (at the nearest 100 g) lightly clothed (without shoes) at every visit. Body mass index (BMI) was calculated as the body weight (kg) divided by the square of the height (m).

Bioelectrical impedance analysis (BIA) was performed at every visit. Standard, 50 kHz frequency, whole-body tetrapolar measurements were obtained from a pair of injector and detector electrodes on hand and foot [4,5]. Impedance vector components, Resistance (R) and Reactance (Xc) were measured and recorded.

### Inclusion criteria

18 to 45 yr old women, sexually active and at risk of pregnancy, having regular menstrual cycle occurring every 25 – 35 days (except for women using an implant), not pregnant, with a normal Pap smear within the previous 12 months, who agreed to use only the assigned study drug as contraception during the study for up to 6 cycles. All subjects signed the informed consent form.

### Exclusion criteria

Known history or presence of disorders commonly accepted as contraindications to hormonal contraceptives including presence or history of venous or arterial thrombosis, migraine with focal aura, known or suspected carcinoma of the breast or of the endometrium, liver disorders, undiagnosed abnormal genital bleeding, alcohol or other substance abuse, oily, irritated, or damaged skin at all potential sites of application.

### Study population

Two hundred and seven subjects were enrolled, underwent the screening visit with planned investigations for body composition analysis and used at least one contraceptive patch (ITT population). Twenty five subjects were excluded from statistical analysis: one obese subject with a body weight of 110 kg and a BMI of 38 kg/m^2^, 9 subjects with missing impedance measurements, and 15 subjects with technical error in bioimpedance measurement (Xc/H component > 60 Ohm/m with normal R/H component), e.g. cream film on the skin of hand and foot. Statistical analysis was performed on 182 subjects at T0. These subjects were 29 yr old (18 to 45 yr), with BMI 21.8 kg/m^2 ^(16 to 31 kg/m^2^), and height 163 cm (147 to 180 cm). At the screening visit, subjects provided information about their previous method of contraception (in the 3 months before the study entry). None was declared by 92 subjects, oral contraceptives by 40 subjects, barrier methods by 41 subjects, vaginal ring by 4 subjects, withdrawal method by 4 subjects, and natural family planning method by 1 subjects.

During the study, compliance was remarkably good with over 93% of the cycles with a perfect compliance and a mean subject compliant score of 90%.

Twenty-one subjects failed to complete the study (3 at T1, 8 at T3, and 10 at T6), leaving for the analysis 179 subjects at T1, 171 at T3 and 161 at T6. The reasons for study discontinuation were not related either to body weight gain or to fluid retention (one pregnancy, failure to return n = 2, adverse reactions n = 8, subject's request unrelated to study events n = 7, protocol violation n = 3). Adverse events that led to withdrawal from the study were: headache, spotting, breast pain, localised skin reactions (n = 4) and libido reduction.

#### - Conventional BIA

TBW estimate (liters) using the impedance measurement at the fixed frequency of 50 kHz was calculated with the sex-specific, regression equation that have been recently validated in a large population by Sun et al. The equation has a standard error of the estimate of 2.6 L (bias 0.3 L) and is recommended for healthy females with a normal and fixed hydration of soft tissues (73%) [15]:

TBW (L) = 3.747 + 0.450 H^2^/R + 0.113 Weight (kg), where H is the subject's height

#### - Vector BIA

Bioelectrical impedance vector analysis (BIVA or vector BIA) was performed with the *RXc graph *method [2,3]. An impedance measurement made at 50 kHz is considered a bivariate, gaussian, random vector (Z) with two correlated components, R and Xc normalized by the subject's height (Z/H = (R/H, Xc/H), in Ohm/m). In contrast with regression equations of conventional BIA, direct measurements are considered without assumptions. In an *RXc mean graph*, mean impedance vectors are plotted as arrows with their 95% confidence ellipses in the R-Xc plane. Overlapping 95% confidence ellipses indicate no significant difference in vector position (not significant Hotelling's T^2 ^test). Separate confidence ellipses indicate a significant (P < 0.05) vector displacement (significant Hotelling's T^2 ^test). Vector BIA only needs to take care of the measurement error (2–3%) and of the biological variability of subjects in any clinical condition. Body composition is evaluated through patterns of vector position and displacement [2-4,16]. In short, vector lengthening or shortening in the R-Xc plane, due to an increase or decrease in both components R/H and Xc/H is associated with a decrease or increase, respectively, in soft tissue hydration. Recently, this basic Vector BIA pattern has been validated in pregnancy using deuterium dilution as reference method [4].

### Statistical methods

The programs of the statistical package SPSS (ver. 15, Chicago, IL) were used for standard calculations, including one-way analysis of variance (ANOVA), Student's t test for paired data, and linear correlation coefficient r. Vector analysis was performed with *BIVA software *(Piccoli A, Pastori G: BIVA software. Department of Medical and Surgical Sciences, University of Padova, Padova, Italy, 2002. Available at E-mail: apiccoli@unipd.it) that allowed drawing of 95% confidence ellipses and statistical testing with the Hotelling's T^2 ^test for paired and unpaired data [17] A test P level of less than 0.05 was considered as statistically significant.

## Results

The aim of the study was to test whether a 6-month treatment with the ethinylestradiol/norelgestromin contraceptive patch induced changes in body composition, particularly in body fluid volume.

Average values of body weight, impedance vector components, and estimates of TBW at T0, T1, T3, and T6 are reported in Table 1. Body weight increased little but significantly over time: by 0.26 kg (0.4%) after 1 month, 0.42 kg (0.7%) after 3 months, and 0.64 kg (1.1%) after 6 months. TBW significantly increased by 0.49 L (1.6%) after 3 months, and by 0.51 L (1.7%) after 6 months. After 3 months, the estimated TBW increase (0.49 L) was greater than the body weight increase (0.42 kg), likely due to the high standard error of the estimate of BIA prediction equation [4,15]. The correlation between body weight changes and TBW changes at T1, T3, and T6 versus T0 was low and not significant (0.17 < r < 0.18).

**Table 1:** Distribution of body composition parameters over time

		Body weight, kg		R/H, Ohm/m		Xc/H, Ohm/m		TBW, L	
Visit	n	M	SD	M	SD	M	SD	M	SD

Screening visit, T0	182	58.1	8.1	373.6	53.6	39.8	6.5	30.4	3.6
After 1 month, T1	179	58.4	8.2	370.6	54.5	39.9	5.8	30.7	3.9
After 3 months, T3	171	58.4	8.1	369.1	58.0	39.3	6.0	30.8	4.6
After 6 months, T6	161	58.6	7.9	368.6	57.1	38.9	5.6	30.9	4.3
T1-T0	179	0.26	1.34	-1.92	25.7	0.22	5.1	0.19	1.49
P(t)		0.01		ns		ns		ns	
T3-T0	171	0.42	1.76	-4.89	30.7	-0.51	5.5	0.49	2.40
P(t)		0.002		0.04		ns		0.008	
T6-T0	161	0.64	1.94	-6.11	31.2	-1.11	6.5	0.51	2.07
P(t)		<0.001		0.01		0.03		0.002	

T0, T1, T3, T6 = screening visit, after 1 month, 3 months, and 6 months

T1-T0, T3-T0, T6-T0 = change in parameters after 1 month, 3 months, and 6 months

t = Student's t test for paired data

**Figure 1:**
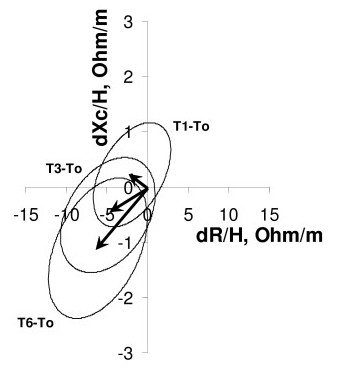
**Impedance vector displacements after 1 month, 3 months and 6 months with their 95% confidence ellipses**. Impedance vector displacements (arrows) after 1 month (T1-T0), 3 months (T3-T0) and after 6 months (T6-T0) with their 95% confidence ellipses. The vector displacement after 6 months is significantly different from zero (P < 0.05, 95% confidence ellipse not overlapping zero), due to a decrease in both components R/H and Xc/H. R is resistance, Xc reactance, H height, and d difference with respect to the screening visit T0.

Vector analysis of impedance documented a significant vector displacement only after 6 months, when both R/H and Xc/H significantly decreased with respect to T0, as shown by the 95% confidence ellipse of mean differences that didn't overlap zero (Figure 1) (P < 0.05, (Hotelling's T^2 ^test for paired data). In agreement with the basic pattern of vector analysis, the decrease in both vector components indicates an increase in soft tissue hydration [2-4,16].

**Figure 2:**
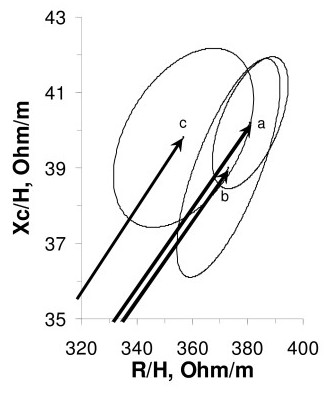
**Mean impedance vectors with their 95% confidence ellipses at the screening visit, by previous contraception method**. Mean impedance vectors with their 95% confidence ellipses at the screening visit. Labels to the vectors represent three groups according to the previous contraception method, vector a from 92 subjects following no method, vector b from 40 subjects taking oral contraceptives, and vector c from 41 subjects using barrier methods. Overlapping confidence ellipses indicate a comparable distribution of vectors among groups.

**Figure 3:**
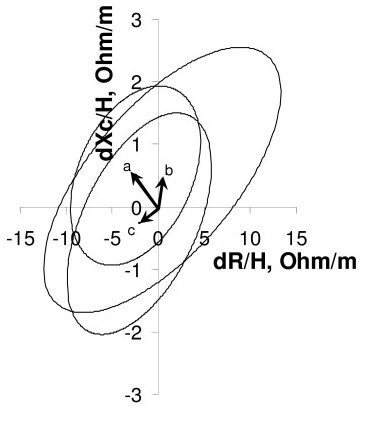
**Impedance vector displacements after 1 month, by previous contraception method**. Impedance vector displacements (arrows) after 1 month. Labels to the vectors represent three groups according to the previous contraception method (as in Figure 2). Overlapping confidence ellipses indicate a comparable change of vectors in the three groups.

We evaluated the distribution of body weight, impedance vector, and TBW by previous contraception method in 173 subjects forming the groups "no method", "oral contraceptives" and "barrier methods" (Table 2). At T0, body weight and impedance vector did not differ among the three previous contraception methods. At baseline, there was a significant difference in TBW among the three methods, due to a greater estimate in the group previously using barrier methods (31.7 L) compared to the group using no method (29.8 L). This result is in contrast with the similar impedance measurements obtained in the three groups at T0 (Figure 2). Also after 1 month with transdermal patch, TBW differed among groups due to the same inequality (32 L in barrier vs 30.1 L in none). However, after 1 month there was neither significant difference in changes in body weight, impedance vector, nor TBW among previous contraception methods (Table 2). Indeed, as shown in Figure 3, the three 95% confidence ellipses of impedance vector displacements after 1 month were overlapping, suggesting no difference in body composition changes among groups.

**Table 2:** Distribution of body composition parameters by previous contraceptive method

		Body weight, kg		R/H, Ohm/m		Xc/H, Ohm/m		TBW, L	
Visit	n	M	SD	M	SD	M	SD	M	SD

T0/CM = 1	92	57.3	8.1	380.9	52.5	40.2	6.7	29.8	3.5
T0/CM = 2	40	59.4	8.4	373.0	45.7	39.0	7.1	30.8	3.4
T0/CM = 3	41	59.2	8.1	356.8	62.8	39.8	5.9	31.7	4.1
P(ANOVA)		ns		ns		ns		0.02	
T1/CM = 1	89	57.8	8.2	376.4	51.5	40.6	5.7	30.1	3.8
T1/CM = 2	40	59.2	8.4	373.4	47.0	39.5	6.4	30.8	3.5
T1/CM = 3	41	59.4	8.0	354.8	67.5	39.5	5.4	32.0	4.6
P(ANOVA)		ns		ns		ns		0.04	
T1-T0/CM = 1	89	0.40	1.42	-2.48	26.6	0.50	5.4	0.21	1.58
T1-T0/CM = 2	40	-0.18	1.12	0.42	31.6	0.43	5.2	-0.02	1.56
T1-T0/CM = 3	41	0.28	1.19	-2.06	19.5	-0.27	4.4	0.29	1.37
P(ANOVA)		ns		ns		ns		ns	

CM = previous contraceptive method: 1 none, 2 oral contraceptive, 3 barrier method

ANOVA = Analysis of variance

T0, T1 = screening visit, after 1 month

T1-T0 = change in parameters after 1 month

Arterial blood pressure did not significantly change over time. Systolic blood pressure was 113 mmHg (SD 10) at T0 and 114 mmHg at T6 (SD 10); diastolic blood pressure was 71 mmHg at T0 (SD 7) and 71 mmHg at T6 (SD 7).

## Discussion

In this study we tested whether a 6-month treatment with the ethinylestradiol/norelgestromin contraceptive patch induced changes in body composition, particularly in body fluid volume. After a treatment of 6 months we documented a small but statistically significant increase in body weight, 0.64 kg (1.1%), that has been due to an increase in soft tissue hydration of similar amount (TBW 0.51 L, 1.7%). This quantity of fluid is within the physiological range of interstitial gel’s hydration fluctuations  [18,19]. In fact, we interpreted this small increase in soft tissue hydration as a physiological change in the interstitial gel fluid which may represent an adaptation to the estrogen component of the patch. As gel fluids are not free to move inside vessels, it does not increase the blood volume [18,19]. Consistently, we did not document any effect on blood pressure.

Therefore, the changes in body weight occurred in our study are clinically not relevant. None of our women dropped out for weight gain, fluid retention or perceptions of them. In this study, women's acceptability and satisfaction with the contraceptive patch were high, their compliance remarkably good and, after 6 months, the majority of subjects indicated that they would continue using the patch.

According to Guyton's theory [18], fluid overload is detectable as apparent edema when interstitial pressure becomes positive due to an increase of interstitial fluid volume above 30% (meaning increase > 4–5 kg body weight, or > 12% TBW). In a normal subject (40 liters of TBW), the interstitial fluid volume is 15 liters in a gel form and with a negative interstitial pressure of – 2 mmHg, the blood volume is 5 liters, and tissue impedance is normal. When the interstitial fluid pressure rises above zero most of the extra fluid is free fluid allowing the appearance of pitting edema and shortening the impedance vector due to a decrease of both vector components. Hence, vector migration on the R-Xc plane can be used in monitoring tissue hydration before the appearance of clinical signs of fluid overload [16,19].

Also a recent review on transdermal hormonal contraception concludes that patch users generally experience minimal, clinically not relevant, changes in body weight [14]. In a recently published meta-analysis on 47 articles [13] there was no evidence supporting a causal association between combined oral contraceptive or a combined skin patch and weight gain. Interestingly, in 15 studies that used ethinylestradiol 20 μg in one arm of treatment, 4 considered weight change at 3, 4, or 6 months and found a range from -0.11 to 0.88 kg (compared to our 0.64 kg) [20-23]. Three studies considered mean body mass percentage change (1.1% as in our population). Other studies evaluated the odds ratio of a relevant weight gain, that was set to > 2 kg of weight or >5%. Analyzing the daily weights of 128 women in treatment with oral contraceptives, Rosenberg found that there were minor fluctuations, clinically not relevant, of body weight during each cycles, confirming that weight gain is a myth or a misperception [24].

We used BIA methods to establish the nature of the body weight change. Clinical validation studies established that a change in the body weight due to a fat mass change in the order of 10 kg does not displace the impedance vector. In contrast, a change in the body weight due to a fluid volume change in the order of 0.5 L is detected as a vector displacement parallel to the major axis of vector's tolerance and confidence ellipses (i.e. change in both R/H and Xc/H components) [2,16,25]. Conventional BIA equations are functions not only of R but also of the body weight. Any body weight change will yield a change in TBW and in both fat and fat-free mass due to the assumption of fixed hydration of soft tissues [25]. With Vector BIA we documented the same pattern of increased hydration of soft tissues as found by Lukaski in pregnancy in a larger scale of hydration [4]. With conventional BIA we estimated an increase in TBW that was in the expected range for normal tissue hydration (TBW = 73% of 0.64 kg soft tissue, i.e. 0.47 L). After 1 month with transdermal patch there was no significant difference in changes in body weight, impedance vector, nor TBW among previous contraception methods (Table 2).

Although we are more confident in Vector BIA as a tool for body composition in any clinical condition [16,19], we estimated TBW for the sake of comparison with the scanty literature, where impedance values are not reported but comparable TBW values were found [26,27]. We found conflicting indications from TBW vs Vector BIA, probably due to the high standard error of BIA prediction equation estimates [4,15]. For instance after 3 months, the estimated TBW increase (0.49 L) was greater than the body weight increase (0.42 kg); at T0 there was a significant difference in TBW among the three previous contraception methods, although body weight and impedance vector did not differ among the three methods (Fig. 2). This may occur because the prediction error of BIA equations is the sum of five errors, namely the impedance measurement error, the regression error against the reference method, the intrinsic error of the reference method, the electric-volume model error, and the biological variability among subjects. *Vector BIA *only needs to take care of the measurement error and of the biological variability of subjects. Hence no change in impedance means no change in hydration independent of the body weight change [16,19].

However, estimates of TBW and of derived compartments (fat and fat-free mass) with BIA prediction equations are not useful in the individual subject due to the high prediction error [4,5,15]. Even weaker is the validity of the estimation of intra- and extracellular fluid with multifrequency BIA [16,28,29] due to tissue anisotropy (part of the current flow is intracellular at low frequencies). For these reasons we used the standard, 50 kHz frequency current (which has the best signal-to-noise ratio) and the Vector BIA which is very sensitive to biological variation of measurements without the need of regression equations [4,16].

The sensitivity of Vector BIA in detecting changes in soft tissue hydration of less than 1 L (0.64 kg of body weight) could help clinicians in routine, objective monitoring of the patients’ body fluid volume, particularly with patients who have the perception of increased body fluid volume of patients, particularly of those with the perception of increased body fluid volume.

Our findings were documented in a "real world" population by using broad criteria for age (18 to 45 yr) and BMI (16 to 31 kg/m^2^). Validity of results can therefore be extended to the routine clinical setting, while this is not possible for other studies on extremely selected subjects (e.g. age 18 to 38 yr, and BMI 21 to 25 kg/m^2^) [30]. Finally, even if our women had been monitored only for 6 months, we are quite confident that we would have obtained more or less the same results with a longer study period. As for body weight, in other published studies the patch was used for up to 13 cycles showing no significant signs of weight gain. As for body composition, other published trials evaluating the effect of oral contraceptives on body water and body fat lasted for a maximum of 6 months [14,26,27,30,31].

An important limitation of the study is the lack of a control group. However, a randomised controlled trial comparing a combined contraceptive method with a placebo or non-hormonal method for contraception raises ethical issues. As a rough surrogate we classified subjects according to their contraception method used in the three months before study entry (no method versus oral contraceptives versus barrier method) and we compared changes in body composition at entry and after 1 month of treatment. With Vector BIA we proved that there was neither a baseline difference nor a differential change in body composition associated with contraception methods used in the three months before.

In any case, present findings will prompt a comparative, or, if ethically acceptable, a placebo/non-hormonal controlled trial with a longer follow up period.

## Conclusion

After 6 months with the ethinylestradiol/norelgestromin contraceptive patch we did not find clinically relevant changes either in body weight or composition, reinforcing observation from other published studies. The minimal increase in body weight, less than 1 kg, could be attributed to an adaptive interstitial gel hydration, physiological as confirmed by the lack of any effect on blood pressure. This could be useful in increasing women's choice, acceptability and compliance of the ethinylestradiol/norelgestromin contraceptive patch. However, further studies, including comparative or controlled trials with a longer follow up period, will help have more robust data concerning the effect of hormonal contraception on body composition.

## Competing interests

The sponsor was Janssen-Cilag EMEA and the funding company was Janssen-Cilag Italy. One author, Silvia Marelli, belongs to the Medical Affairs Department of Janssen-Cilag Italy. The other authors declare that they have no competing interests.

## Authors' contributions

AP performed statistical analysis, contributed to data interpretation and drafted the manuscript; PGC made substantial contributions to conception and design of body composition analysis, was the Principal Investigator of the Italian coordinating site and contributed to data interpretation and critical revision of the manuscript; CN, SR and VB were the Principal Investigators of three Italian sites, gave important contributions to data interpretation and manuscript revision; SM was the Local Trial Coordinator of the study in Italy and was involved in drafting the manuscript and revising it critically for important intellectual content.

All authors have read and approved the final manuscript.
